# Molecular Characterisation of Trimethoprim Resistance in *Escherichia coli* and *Klebsiella pneumoniae* during a Two Year Intervention on Trimethoprim Use

**DOI:** 10.1371/journal.pone.0009233

**Published:** 2010-02-16

**Authors:** Alma Brolund, Martin Sundqvist, Gunnar Kahlmeter, Malin Grape

**Affiliations:** 1 Department of Microbiology, Tumor and Cell Biology (MTC), Division of Clinical Microbiology, Karolinska Institutet, Stockholm, Sweden; 2 Department of Clinical Microbiology, Central Hospital, Växjö, Sweden; 3 Division of Infectious Diseases, Department of Medical Sciences, Uppsala University, Uppsala, Sweden; 4 Division of Clinical Bacteriology, Department of Medical Sciences, Uppsala University, Uppsala, Sweden; Massachusetts General Hospital, United States of America

## Abstract

**Background:**

Trimethoprim resistance is increasing in *Enterobacteriaceae*. In 2004-2006 an intervention on trimethoprim use was conducted in Kronoberg County, Sweden, resulting in 85% reduction in trimethoprim prescriptions. We investigated the distribution of *dihydrofolate reductase* (*dfr*)-genes and integrons in *Escherichia coli* and *Klebsiella pneumoniae* and the effect of the intervention on this distribution.

**Methodology/Principal Findings:**

Consecutively isolated *E. coli* (n = 320) and *K. pneumoniae* (n = 54) isolates phenotypicaly resistant to trimethoprim were studied. All were investigated for the presence of *dfrA1, dfrA5, dfrA7, dfrA8, dfrA12, dfrA14, dfrA17* and integrons class I and II. Isolates negative for the seven *dfr*-genes (n = 12) were also screened for *dfr2d*, *dfrA3*, *dfrA9*, *dfrA10*, *dfrA24* and *dfrA26*. These genes accounted for 96% of trimethoprim resistance in *E. coli* and 69% in *K. pneumoniae*. The most prevalent was *dfrA1* in both species. This was followed by *dfrA17* in *E. coli* which was only found in one *K. pneumoniae* isolate. Class I and II Integrons were more common in *E. coli* (85%) than in *K. pneumoniae* (57%). The distribution of *dfr*-genes did not change during the course of the 2-year intervention.

**Conclusions/Significance:**

The differences observed between the studied species in terms of *dfr-*gene and integron prevalence indicated a low rate of *dfr-*gene transfer between these two species and highlighted the possible role of narrow host range plasmids in the spread of trimethoprim resistance. The stability of *dfr*-genes, despite large changes in the selective pressure, indirectly suggests a low fitness cost of *dfr*-gene carriage.

## Introduction

The increase in antibiotic resistance is a threat to global health [1]. Trimethoprim is commonly used in the treatment of urinary tract infections (UTI) in all parts of the world [2]. However, already soon after the introduction of the drug, trimethoprim resistance was reported in several species [3] and are now in unselected UTI materials at levels of 15–65% in *E. coli* [4], [5], [6]. This leads to treatment failure and increasing workload in primary healthcare and trimethoprim containing antibiotics are thus questioned as first line therapy [7].

Resistance to trimethoprim is caused by modifications in the target enzyme dihydrofolate reductase (dfr) encoded by *dfr-*genes. So far 30 *dfr*-genes are described and they are usually associated with integrons [3]. Integrons are integrated in transposons predominantly located on plasmids and can insert, excise and express mobile gene cassettes, often antibiotic resistance genes [8]. This results in an efficient horizontal spread of antibiotic resistance between bacteria [8], [9], [10]. Only few studies have investigated the epidemiology and frequency of the different *dfr-*genes and the relationship to integrons and other resistance determinants in *E. coli* [9], [11], [12]. In *K. pneumoniae* the distribution of *dfr-*genes is not known.

A two year prospective intentional intervention on the use of trimethoprim was performed in Kronoberg County, Sweden, from October 1^st^, 2004 to September 30^th^, 2006. A drastic and sustained 85% reduction in the use of trimethoprim and trimethoprim-sulphamethoxazole was achieved at county level. A corresponding increase was seen in the use of nitrofurantoin, pivmecillinam and to some extent ciprofloxacin. The effect on trimethoprim resistance was marginal[13] explained by the low fitness cost of trimethoprim resistance observed and the high level of associated resistance in trimethoprim resistant isolates [13]. During 4 years including the intervention all urinary tract isolates of *Enterobacteriaceae*, both hospital and community acquired, were stored frozen at −70°C. Using these, we investigated the distribution of 13 *dfr-*genes and class I and II integrons in trimethoprim resistant *E. coli* and *K. pneumoniae*. We also investigated the associated resistance rates in these isolates and the findings are discussed in relation to the epidemiology of trimethoprim resistance within and between the two species.

## Materials and Methods

### Material

Three hundred and twenty (320) consecutively collected trimethoprim resistant *E. coli* isolates during three time periods were identified. The periods were: four months before the intervention (June to September 2004 (n = 106)), one year into the intervention (October to December 2005 (n = 105)) and the last four months of the intervention (June to September 2006 (n = 109)). Within each of the time periods duplicate patient samples were excluded. Fifty four (54) consecutive trimethoprim resistant *K. pneumoniae* isolates from June 2004 to September 2007, reflecting the relative number of isolates of the respective pathogens in UTI, were retrieved. Isolates were from urinary samples from hospitals and community and from all age groups.

### Methods

#### Susceptibility testing

*Escherichia coli* and *K. pneumoniae* were identified according to standard procedures used in the Department of Clinical Microbiology, Växjö, Sweden, at the time of the study. Susceptibility testing was performed using disc diffusion on IsoSensitest Agar (Oxoid, Basingstokes, UK) according to the Swedish Reference Group on Antibiotics (SRGA) guidelines (http://www.srga.org/). All isolates were tested with the following antibiotic discs (Oxoid, Basingstokes, UK) mecillinam 10 µg, ampicillin 10 µg, cefadroxil 30 µg, trimethoprim 5 µg, nitrofurantonin 100 µg and nalidixic acid 30 µg. To obtain the most sensitive detection of antibiotic resistance, the resistant and susceptible populations were identified by calculating the epidemiological cut-off values by using the Normalized interpretation method (NRI) [14]. For mecillinam the SRGA R-breakpoint used at the time of isolation was applied to avoid false associations to TMP resistance due to concomitant AMP resistance affecting the MEC MIC only marginally (i.e. MIC≤8) and considered not to be due to specific mecillinam resistance mechanisms (Kahlmeter unpublished data). Trimethoprim resistance was further analysed using E-test (bioMérieux, Solna, Sweden).

#### PCR

DNA lysates were prepared by resolving a loop full (1 µl) of bacteria in 100 µl DNase and RNase free water (Sigma-Aldrich, Stockholm, Sweden). The mixture was boiled for 2 minutes and the tubes were centrifuged at 13000 rpm for 2 minutes. The supernatant was stored in −20°C and used as DNA template. All isolates, both *E. coli* and *K. pneumoniae*, were first screened for the five most prevalent resistance genes reported in trimethoprim resistant *E. coli*; *dfrA1, dfrA5, dfrA7, dfrA12* and *dfrA17* [9], [11] using a previously published real-time multiplex PCR protocol [15]. In addition *dfrA8* and *dfrA14* was screened for among all isolates. In isolates negative to these genes (n = 12) *dfr2d*, *dfrA3*, *dfrA9*, *dfrA10*, *dfrA24* and *dfrA26* were analysed using separate PCRs. Internal controls with primers for 16S rDNA was used as template control [16]. All isolates were in addition analyzed for the presence of integron class I and II, which are the most common integrons associated with *dfr-*genes [8]. The screening of *dfr*-genes and integrons class I and II was performed using simplex PCRs, described by Grape et al [17]. The PCR for integron class I detection was performed using two sets of primers to avoid false negative results due to modifications in the 3′CS region. A PCR product with either of these primer pairs was considered a positive result. Previously unpublished primers are described in Table 1.

**Table 1:** Primers not previously published used in the PCR screenings.

Primer	Locus	Sequence (5′-3′)	Annealing temperature (C)	Product size (bp)	Reference
dfrA14-f	5′ dfrA14	CTG CGA AAG CGA AAA ACG GCG	55	376	This study
dfrA14-r	3′ dfrA14	GGA ATA CTC GGG AAG AAA ACA	55		This study
dfrA24-f	5′ dfrA24	CGT TGC TGC TAC TGA GAA CG	54	158	This study
dfrA24-r	Mid dfrA24	TGC GGT CTT TCA GAG GAC TT	54		This study
dfrA26-f	5′ dfrA26	GGT AAC GCG TCA ACA AGG TT	56	190	This study
dfrA26-r	3′ dfrA26	GGC GTG TAC TTC GGT GAG AT	56		This study

#### Semi-random PCR

Semi random PCR [18] was performed on three *E. coli* isolates where the PCR only yielded an amplicon corresponding to the 5′CS (IntI1) of integron class I. Primers for identification of resistance cassettes integrated in integron class I were designed targeted at IntI1; 51 bp and 27 bp respectively from the cassette region (Table 2).

**Table 2:** Primers not previously published used in the semi random PCR and sequence analysis.

Primer	Locus	Sequence (5′-3′)	Annealing temperature (C)	Product size (bp)	Reference
IntI1-f	5′ CS class 1 integrons 51 bp from casette region	TAC GCC GTG GGT CGA TGT TTG ATG	64	Depend on where Arb1 or 6 binds	[24]
IntI1-s	5′ CS class 1 integrons 27 bp from casette region	TTA TGG AGC AGC AAC GAT GT	50	Depend on where Arb1 or 6 binds	This study

#### Sequence analysis

Sequence analysis was performed on the semi-random PCR amplicons as well as on two isolates where we could only detect a complete integron class I structure (5′CS-3′CS). PCR products (50 µl reactions) were purified using Jet Quick PCR Product Purification Spin kit (Genomed, Löhne, Germany) or, in case of unspecific PCR products, extracted from electrophoresis gel by using the Jet Quick Gel Extraction Spin kit (Genomed, Löhne, Germany). Sequencing reactions were performed by using the ABI Prism Big Dye Terminator Cycle Sequencing kit v. 3.1 (Applied Biosystems, Stockholm, Sweden) according to manufacturer's instructions. Sequence data was analysed with the Sequencher software v. 4.1.4 (Gene Codes Corporation, Ann Arbor, MI).

### Ethics

As this study was based on bacterial isolates collected from routine samples, approval from ethics committee was not necessary. Neither was there any need for informed consent procedures as no extra sampling was performed and no personal data was stored in relation to the isolates.

### Statistics

|^2^-test was used for comparing *dfr*-gene prevalence and distribution in *E. coli* and *K. pneumoniae*.

## Results

### *dfr-*Genes and Integrons

The total prevalence of the 13 analysed *dfr-*genes was 96% (n = 308) in *E. coli* and 69% (n = 38) in *K. pneumoniae*. The integron prevalence (both integron class I and II) was 85% (n = 273) and 57% (n = 31) in *E. coli* and *K. pneumoniae*, respectively. These differences were statistically significant (p<0.001). The most common trimethoprim resistance gene in both investigated species was *dfrA1*. The second most frequent *dfr-*gene in *E. coli, dfrA17*, was rare in *K. pneumoniae* - it was found in only one isolate. The genes *dfr2d* and *dfrA24* were found only in *E. coli* and genes *dfrA3* and *dfrA9* only in *K. pneumoniae*. Four *E. coli* isolates and one *K. pneumoniae* isolate carried two *dfr*-genes. The *dfrA1* gene was present together with either *dfrA5*, *dfrA7*, *dfrA14* or *dfrA17* in *E. coli* and together with *dfrA8* in *K. pneumoniae*.

The prevalence of each of the 13 different *dfr-*genes and integrons class I and II found in the *E. coli* and *K. pneumoniae* isolates are presented in Table 3. No major shifts in the *dfr-*gene/integron distribution were seen in the *E. coli* isolates during the intervention. The *K. pneumoniae* collection was too small for statistically significant changes to be detected.

**Table 3:** No. of isolates positive for the investigated *dfr-*genes and integrons in *E. coli (n = 320)* and *K. pneumoniae (n = 54)* collected at time periods before and during the trimethoprim intervention.

	*E. coli*				*K. pneumoniae*
	Pre intervention n = 106	Mid intervention n = 105	Post intervention n = 109	Total n = 320 (%)	Total n = 55 (%)
*dfrA1*	36	41	33	110(34)	8 (15)
*dfr2d*	1	0	0	1(0.3)	0
*dfrA3*	0	0	0	0	1 (2)
*dfrA5*	17	17	18	52(16)	7 (13)
*dfrA7*	3	7	5	15(5)	1 (2)
*dfrA8*	3	3	8	14(4)	7 (13)
*dfrA9*	0	0	0	0	1 (2)
*dfrA10*	0	0	0	0	0
*dfrA12*	6	2	6	14 (4)	7 (13)
*dfrA14*	9	4	6	19 (6)	5 (9)
*dfrA17*	26	29	27	82 (26)	1 (2)
*dfrA24*	1	0	0	1 (0.3)	0
*dfrA26*	0	0	0	0	0
Integron 1	75	74	82	230 (72)	29 (53)
Integron 2	10	19	14	43 (13)	2 (4)

In 83 *E. coli* isolates the integron screenings only detected the integrase gene (5′CS of the integron class I) as opposed to the whole integron structure (5′CS-3′CS). In *K. pneumoniae* the corresponding figure were 14 isolates. In the *E. coli* collection there were eight isolates where the class I integrase but no *dfr*-genes was detected. Semi-random PCR was used on these eight isolates to seek for *dfr*-genes downstream from this element. This resulted in the detection of five isolates with *dfrA14*. Since *dfrA14* and *dfrA8* appeared to be present more than we had expected we decided to screen the whole collection of *E. coli* as well as *K. pneumoniae* for this gene. In two *E. coli* isolates PCR detected the whole integron class I structure but no *dfr*-genes. The integron class I was then sequenced and found to include *dfrA1*. The *dfrA1*-gene in these isolates had mutations in the reverse primer binding site, which is probably why they were not found with the multiplex PCR analysis. After these analyses 10 out of 320 *E. coli* isolates ended up having an unknown genetic background of trimethoprim resistance. Seven of these were integron class I and II negative. In the *K. pneumoniae* collection 17 out of 54 isolates were negative for the *dfr-*genes analysed and 14 of these were negative for the integrons investigated. The relationship between the *dfr-*genes studied and integron prevalence in *E. coli* and *K. pneumoniae* is described in Table 4. Class II integrons are known to be conserved and always includes *dfrA1*, which was also the case in these investigated isolates. No integrons were detected in isolates positive for *dfr2d* and *dfrA9*.

**Table 4:** The relationship between *dfr-*genes and integron class I and II in *E. coli* (n = 320) and *K. pneumoniae* (n = 54).

*E. coli*	No.	No.	*K. pneumoniae*	No.	No.
	Igr1	Igr2		Igr1	Igr2
*dfrA1* a	61	44	*dfrA1* b	5	2
*dfr2d*	0	0	*dfr2d*	0	0
*dfrA3*	0	0	*dfrA3*	1	0
*dfrA5*	49	0	*dfrA5*	7	0
*dfrA7*	15	0	*dfrA7*	1	0
*dfrA8*	1	0	*dfrA8*	1	0
*dfrA9*	0	0	*dfrA9*	0	0
*dfrA10*	0	0	*dfrA10*	0	0
*dfrA12*	12	0	*dfrA12*	7	0
*dfrA14*	8	0	*dfrA14*	4	0
*dfrA17*	81	0	*dfrA17*	1	0
*dfrA24*	1	0	*dfrA24*	0	0
*dfrA26*	0	0	*dfrA26*	0	0
All *dfr*+	228	44	All *dfr*+	27	2

a Including 10 isolates positive for both Igr1 and 2.

b Including one isolate positive for both Igr 1 and 2.

### Resistance Phenotypes

All isolates were tested for susceptibility to antibiotics commonly used in the treatment of UTI. As expected, the prevalence of integrons was high in the material and the probability of integron carriage increased with the number of resistance determinants (Table 5 and 6). High levels of associated resistance have been described previously in trimethoprim resistant isolates [19]. In the present material ampicillin resistance was 78% and nalidixic acid resistance was 26% in trimethoprim resistant *E. coli*. Most *dfr*-genes were equally associated to these phenotypic resistances but *dfrA8* was only associated with ampicillin resistance in *E. coli*.

**Table 5:** No. of *E. coli* (n = 320) isolates resistant to trimethoprim only and in combination with other antibiotics and in relation to the presence of integrons.

Resistance phenotype	isolates (#)	Integron (%)
TMP (only)	58	71
TMP, AMP	66	61
TMP, NAL	13	69
TMP, AMP, NAL	56	91
TMP, AMP, MEC	10	90
TMP, AMP, CFR	3	100
TMP, NAL, CFR	1	100
TMP, AMP, NAL, CFR	8	100
TMP, AMP, NAL, NIT	5	80
TMP, AMP, NAL, MEC	1	100

TMP = trimethoprim, AMP = ampicillin, NAL = nalidixic acid, MEC = mecillinam, CFR = cefadroxil and NIT = nitrofurantoin.

**Table 6:** No. of *K. pneumoniae* (n = 54) isolates resistant to trimethoprim only and in combination with other antibiotics and in relation to the presence of integrons.

Resistance phenotype	isolates (#)	Integron (%)
TMP, AMP, NIT	27	67
TMP, AMP, NIT, MEC	2	0
TMP, AMP, NIT, NAL	18	39
TMP, AMP, NIT, MEC, NAL	5	40
TMP, AMP, NIT, CFR, MEC	1	100
TMP, AMP, NIT, CFR, NAL	1	100

TMP = trimethoprim, AMP = ampicillin, NAL = nalidixic acid, MEC = mecillinam, CFR = cefadroxil and NIT = nitrofurantoin.

## Discussion

Although common, the epidemiology of trimethoprim resistance in other species than *E. coli* has not been well studied at the molecular level. Recently we performed a highly controlled intervention on trimethoprim use in Kronoberg County, Sweden. Despite a drastic decrease (85%) in trimethoprim consumption the effect on the rate of trimethoprim resistance in *E. coli* was disappointingly marginal [13]. This was explained by co-selection mediated by a high level of associated resistance in trimethoprim resistant isolates and by a low fitness cost conferred by trimethoprim resistance measured *in vitro* in clinical isolates of *E. coli* [13]. The clonal distribution in *E. coli* remained unaffected [13]. The present study shows a lack of effect on the overall distribution of *dfr-*genes (Table 3). It also compares the distribution of *dfr*-genes in *E. coli* and *K. pneumoniae.*

The overall prevalence of the different *dfr-*genes in *E. coli* was in line with earlier studies performed in different clinical and geographical settings by, amongst others, Blahna [9] and Lee. [11] These studies have indicated a very stable distribution of *dfr*-genes over time with *dfrA1* and *dfrA17* as the most prevalent genes. The stability over time and throughout the intervention suggests that the epidemiological fitness cost of the most common *dfr-*genes or of plasmids carrying these genes is very low. The fact that *dfrA8* and *dfrA14* were as common as *dfrA7* and *dfrA12* indicates possible future shifts in the *dfr*-gene distribution. So far *dfrA3*, *dfrA8*, *dfrA9*, *dfrA10* and *dfrA24* have not been reported as cassettes in integron structures [20]. Multiple *dfr*-genes occur but are unusual. The PCR screening performed for *dfrA1*, *dfrA5*, *dfrA7*, *dfrA8*, *dfrA12, dfrA14* and *dfrA17* detected coexistence of two genes in four out of 320 *E. coli* isolates and one out of 54 *K. pneumoniae* isolates. Previous studies also confirm this; in a large European study [9] they found multiple *dfr*-genes in two out of 163 trimethoprim resistant isolates and in a Korean study [11] the same figure was three out of 77 trimethoprim resistant isolates. Based on this we decided to screen for the additional six *dfr*-genes in the trimethoprim resistant isolates that were negative in the multiplex PCR analysis.

The prevalence of *dfr-*genes and integrons in *K. pneumoniae* has not been studied systematically before and was surprisingly different from *E. coli*. The low prevalence of integrons class I and II could suggest other mechanisms at play or that transfer of *dfr*-genes in *K. pneumoniae* does not occur. The genes *dfrA5*, *dfrA8* and *dfrA12* were more common in *K. pneumoniae* than in *E. coli* and *dfrA17*, being the second most frequent *dfr-*gene in *E. coli*, was only found in a single *K. pneumoniae* isolate. Recent studies in mice showed that plasmids carrying varying resistance genes were easily transferred from an *E. coli* donor to *K. pneumoniae* in a mouse model. [21] Our data, showing a pronounced disproportion in the prevalence of integrons and *dfr-*genes in *E. coli* and *K. pneumoniae*, speak to the opposite. This could be because *dfr*-genes are carried on narrow host range plasmids, or are integrated in the chromosomal DNA or because of differences in fitness cost of specific *dfr-*genes in *E. coli* and *K. pneumoniae*. The trimethoprim resistant *K. pneumoniae* were collected over three years, from individuals of all age groups and varying residency. The *K. pneumoniae* isolates were not typed but with this background clonal spread of these isolates seems an unlikely explanation for the observed differences between *E. coli* and *K. pneumoniae*.

Trimethoprim resistance is often associated with other resistance determinants. [8], [9], [17], [22] This gives the possibility for co-selection of trimethoprim resistant strains and plasmids carrying both *dfr-*genes as well as other resistance determinants by the use of other antibiotic classes. In this study no striking difference in the distribution of other resistance determinants in relation to specific *dfr*-genes was seen. The only exception was *E. coli* isolates carrying *dfrA8* (n = 10) where an association exclusively with ampicillin resistance were found. Only one out of ten of these isolates carried an integron class I. As a bright contrast, *dfrA17* was found to be associated with resistance to all investigated antibiotics and 81 out of 82 isolates also carried an integron class I. The association of the *dfr-*genes studied and quinolone resistance is to be explained by co-existence in the same isolates rather than association on fellow plasmids as the frequency of plasmid associated quinolone resistance genes (*qnr* and *aac(6*′*)-Ib-cr*) in our area is very low [23]. These findings suggests some lineages in *E. coli* to be better suited to acquire and stabilize antibiotic resistance determinants and reveals a need for future studies to understand the associations between plasmids, integrons, gene-cassettes and the genetic backbone.

In conclusion, trimethoprim resistance in *E. coli* was explained almost entirely by the 13 investigated *dfr-*genes. These isolates were also to a large extent positive for integrons class I and/or II. The distribution of *dfr-*genes in *E. coli* was in line with earlier published data and remained stable throughout the intervention. In *K. pneumoniae* we observed a low prevalence of the most common *dfr-*genes in *E. coli* and a surprisingly low rate of integron class I and II. This indicates that the exchange of *dfr-*genes between *E. coli* and *K. pneumoniae* may be an uncommon event in man. These results suggest the need for more studies on the genetic context of *dfr*-genes and their location in or association with mobile genetic elements and other resistance genes as well as their association to certain plasmid types. It reminds us that we can not only rely on model organisms, such as *E. coli*, to understand the variety of phenotypic resistance.
